# Chemokines and Chemokine Receptors: New Targets for Cancer Immunotherapy

**DOI:** 10.3389/fimmu.2019.00379

**Published:** 2019-03-06

**Authors:** Valeria Mollica Poeta, Matteo Massara, Arianna Capucetti, Raffaella Bonecchi

**Affiliations:** ^1^Humanitas Clinical and Research Center, IRCCS, Rozzano, Italy; ^2^Department of Biomedical Sciences, Humanitas University, Pieve Emanuele, Italy

**Keywords:** immunotherapy, cancer related inflammation, atypical chemokine receptor, chemokine receptor, chemokine

## Abstract

Immunotherapy is a clinically validated treatment for many cancers to boost the immune system against tumor growth and dissemination. Several strategies are used to harness immune cells: monoclonal antibodies against tumor antigens, immune checkpoint inhibitors, vaccination, adoptive cell therapies (e.g., CAR-T cells) and cytokine administration. In the last decades, it is emerging that the chemokine system represents a potential target for immunotherapy. Chemokines, a large family of cytokines with chemotactic activity, and their cognate receptors are expressed by both cancer and stromal cells. Their altered expression in malignancies dictates leukocyte recruitment and activation, angiogenesis, cancer cell proliferation, and metastasis in all the stages of the disease. Here, we review first attempts to inhibit the chemokine system in cancer as a monotherapy or in combination with canonical or immuno-mediated therapies. We also provide recent findings about the role in cancer of atypical chemokine receptors that could become future targets for immunotherapy.

## Role of Chemokines in Tumors

Inflammation is an essential component of the tumor microenvironment and one of the hallmarks of cancer (1, 2). Chemokines, are a family of small, secreted, and structurally related cytokines with a crucial role in inflammation and immunity (3). They are also key mediators of cancer related inflammation being present at tumor site for pre-existing chronic inflammatory conditions but also being target of oncogenic pathways (4). Initially identified with a prominent role in determining the composition of tumor stroma, they were found able to directly affect cancer cell proliferation and metastasis (5, 6).

### Leukocyte Recruitment

The proper movement of immune cells is orchestrated by the spatial and temporal expression of chemokines. Inflammatory CC (CCL2, CCL3, CCL5) and CXC (CXCL1, CXCL2, CXCL5, CXCL6, and CXCL8) chemokines recruit at the tumor site CCR2^+^ monocytes and CXCR2^+^ neutrophils that differentiate into tumor associated macrophages (TAMs) and tumor associated neutrophils (TANs), exerting pro- or anti-tumoral role (7–10). Some chemokines present at tumor site can modify leukocyte activation, for instance CXCL16 acting on CXCR6 induces macrophage polarization toward a pro-tumoral phenotype in solid tumors (11, 12). CXCL9 and CXCL10 are strongly associated with Th1 immune response by recruiting NK cells, CD4^+^ Th1 and CD8^+^ cytotoxic lymphocytes, which can elicit antitumoral responses (13, 14). Moreover, potent attractant of dendritic cells (DC) are CCL20, CCL5, and CXCL12 (15); CCL21 and CCL19 recruit CCR7^+^ DC but also regulatory T cells (T_regs_) (16, 17). CCL17 and CCL22 acting on CCR4 can directly recruit T_regs_ and Th2 lymphocytes, that promote tumor growth and proliferation (18).

### Angiogenesis

Both CC and CXC chemokines play a critical role in tumor angiogenesis, essential for tumor growth and metastatic spreading (19, 20). CXC chemokines, based on the presence of glutamic-leucine-arginine (ELR) motif at the N-terminal, can be divided in ELR^+^ chemokines with angiogenic and ELR^−^ chemokines with angiostatic effects. CCL2, CCL11, CCL16, CCL18, and CXCL8 promote tumor angiogenesis and endothelial cell survival (21, 22). Moreover, CXCL16 interacting with CXCR6, acts as a potent angiogenic mediator (23). CXCL12 and CCL2 can promote angiogenesis and inhibit apoptosis of endothelial cells by directly binding their receptor (CXCR4 and CCR2, respectively) expressed on tumor vessels or indirectly promoting the recruitment of leukocytes (24, 25). On the contrary, chemokines, such as CCL21 and ELR^−^ chemokines (CXCL4, CXCL9, CXCL10, and CXCL11) inhibit angiogenesis and endothelial cell proliferation (26).

### Tumor Growth and Proliferation

Chemokines produced by tumor itself, cancer-associated fibroblasts and infiltrating leukocytes (27, 28), through the binding of chemokine receptors expressed by tumor cells, directly promote cancer cell proliferation activating different signaling pathways, such as PI3K/AKT/NF-κB and MAPK/ERK pathway (29–31). Additionally, they can promote tumor cell survival by preventing their apoptosis and regulating the balance between pro- and anti-apoptotic molecules (e.g., downregulation of Bcl-2 expression or inhibition of caspase-3 and caspase-9 activation) (32, 33).

### Metastasis

Chemokine receptors expressed by cancer cells promote their migration to metastatic sites (34). Chemokines and chemokine receptors involved in this phenomenon are several: CCR7 mediates the migration of tumor cells to lymph nodes where their ligands, CCL19 and CCL21, are produced (34, 35). The CCR10/CCL27 axis facilitates the adhesion and survival of melanoma cells during metastatic spreading (36). CCL28 promotes breast cancer growth and metastasis spreading through MAPK/ERK pathway (37). Finally the chemokine receptor CXCR5 and its ligand CXCL13 support bone metastases in prostate cancer (38). However, the main player of this process is the CXCL12/CXCR4 axis. In several tumors, CXCR4 expression endows cancer cells with the ability to migrate and metastasize into organs secreting high levels of CXCL12 (6, 39).

## Chemokines in Cancer Therapy

**Figure 1:**
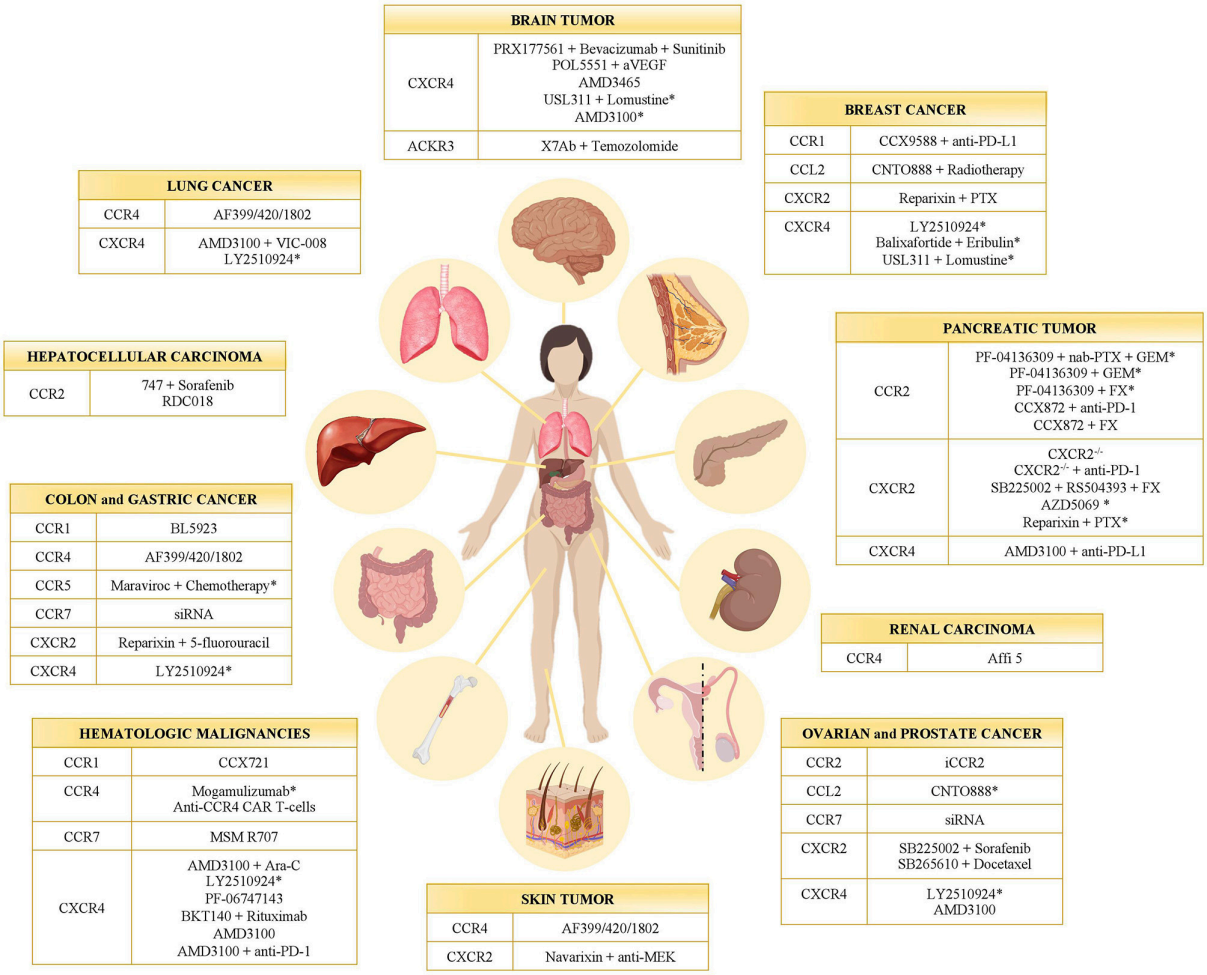
Chemokine receptor inhibitors in cancer. Inhibitors of CC- and CXC-chemokine receptors tested in different tumor types in preclinical models and clinical trials (*). GEM, Gemcitabine; PTX, Paclitaxel; FX, FOLFIRINOX.

Targeting the immune system represents a concrete approach against cancer (40–42). Starting from Coley's toxin development in 1893, many strategies have been set to enhance the antitumor activity of leukocytes (42, 43). Given that chemokines and their receptors have been found involved in several aspects of cancer biology, their possible targeting was evaluated in many preclinical studies and clinical trials (Table 1 and Figure 1). Actually, a monoclonal antibody (anti-CCR4 mAb, Mogamulizumab) and a chemokine receptor inhibitor (CXCR4 antagonist AMD3100) are already in the clinical practice for hematological malignancies (see below).

**Table 1:** Chemokine and chemokine receptor inhibitors in preclinical models and clinical trials.

	**Preclinical models**			**Clinical trials**		
**Target**	**Inhibitor**	**Tumor model**	**References**	**Inhibitor**	**Tumor type**	**References**
CCR1	CCX721	Multiple myeloma	(44, 45)			
	BL5923	Colon cancer liver metastasis	(46)			
	CCX9588 + anti-PD-L1	Breast cancer	(47)			
CCR2	PF-04136309 + GEM	Pancreatic cancer	(48)	PF-04136309+nab- PTX+GEM	Pancreatic cancer	NCT02732938; (49)
	CCX872 + anti-PD-1	Pancreatic cancer	(50)	PF-04136309 + FX	Pancreatic ductal adenocarcinoma	NCT01413022; (51)
	RDC018	Hepatocellular carcinoma	(52)	CCX872 +FX	Pancreatic cancer	NCT02345408; (53)
	747 + Sorafenib	Hepatocellular carcinoma	(54)			
	iCCR2	Ovarian cancer	(55)			
CCL2	CNTO 888 + radiotherapy	Breast cancer	(56)	CNTO 888	Solid tumors	NCT00537368
				CNTO 888	Metastatic prostate cancer	NCT00992186; (57, 58)
CCR4	Anti-CCR4 CAR-T cells	T cell malignancies	(59)	Mogamulizumab	Relapsed/refractory	
	Affi 5	Renal tumor	(61)		ATL	(60)
	AF399/420/1802	Melanoma, lung tumor and CRC	(62)	Mogamulizumab	CTL	NCT01728805; (63)
CCR5	Maraviroc	CRC	(64)	Maraviroc + chemotherapy	CRC	NCT01736813; (64)
CCR7	siRNA	Metastatic CRC and prostate cancer	(65, 66)			
	MSM R707	Metastatic T- ALL	(67)			
CXCR2	Cxcr2^−/−^+ PTX	Breast cancer	(68)	AZD5069	Pancreatic cancer	NCT02583477
	Navarixin + anti-MEK	Melanoma	(69)	Reparixin + PTX	Breast cancer	NCT02370238; (70)
	SB225002 + Sorafenib	Ovarian cancer	(71)			
	Reparixin + 5-fluorouracil	Human gastric cancer	(72)			
	Cxcr2^−/−^	Pancreatic cancer	(73)			
	Cxcr2^−/−^+ anti-PD-1	Pancreatic cancer	(74)			
	SB225002+RS504393+FX	Pancreatic cancer	(75)			
	SB265610 + Docetaxel	Prostate cancer	(76)			
CXCR4	AMD3100 + Ara-C	AML	(77)	AMD3100	Relapsed AML	NCT00512252; (78)
	LY2510924	AML	(79, 80)	LY2510924	CRC, lung, breast, prostate cancer	NCT02737072; (81)
	BKT140 + Rituximab	NHL	(82)	BMS-936564	AML	NCT01120457; (83)
	AMD3465	GBM and Medulloblastoma	(84)	PF-06747143	Hematologic malignancies	NCT02954653
	POL5551 + anti-VEGF	GBM	(85, 86)	USL311 + Lomustine	Solid tumors and GBM	NCT02765165
	AMD3100	Ovarian cancer	(87)	Balixafortide + Eribulin	HER2^−^ metastatic breast cancer	NCT01837095; (88)
	AMD3100 + anti-PD-L1	Pancreatic cancer	(89)	AMD3100	Recurrent GBM	NCI2012-00149;
	AMD3100 + VIC-008	Mesothelioma	(90)			NCI2013-02012
	PRX177561+Bevacizumab+ Sunitinib	GBM	(91)			
ACKR2	Ackr2 ^−/−^	Metastatic breast cancer and melanoma	(92, 93)			
ACKR3	X7Ab + Temozolomide	GBM	(94)			

### CCR1

Inhibition of CCR1 reduces cancer growth and metastatization mainly by targeting myeloid cells. In mouse models of Multiple Myeloma (MM) the CCR1 antagonist CCX721 reduced tumor growth and osteolysis targeting osteoclasts and their precursors (44, 45). The same effect was also given by blocking the CCR1 ligand CCL3 that is highly produced by MM cells (95). In a murine model of colon cancer liver metastasis, the CCR1 antagonist BL5923 inhibited metastasis by limiting the recruitment of immature myeloid cells (46). The CCR1 receptor antagonist CCX9588 was recently used in combination with anti-PD-L1 in a murine model of breast cancer showing a synergistic antitumoral effect by reducing the myeloid infiltrate (47). Due to the fact that CCR1 antagonists did not show adverse effects when used in autoimmune disease patients (96), they are ideal candidates to modulate the myeloid infiltrate in combination treatments.

### CCR2 and CCL2

Interference with the CCL2/CCR2 axis exerts antitumoral activity in many cancers for the reduced recruitment of monocytes with pro-tumorigenic and pro-metastatic activities.

Many data are available in the context of pancreatic tumors. In a preclinical model, the oral CCR2 inhibitor PF-04136309 reduced the number of TAMs and exerted a modest effect on tumor growth when used alone, while it acted synergistically with the chemotherapeutic drug Gemcitabine (GEM) (48). Encouraging results of a Phase Ib/II trial with pancreatic cancer patients, in which PF-04136309 is used in combination with nab-Paclitaxel [(PTX), a nanoparticle albumin-bound formulation of PTX able to induce TAM activation toward an M1 like phenotype] (97), and GEM, were recently published (NCT02732938) (49). The same inhibitor was used in another clinical trial (NCT01413022) performed on borderline resectable or locally advanced pancreatic ductal adenocarcinoma patients in combination with the standard chemotherapy FOLFIRINOX (FX). Preliminary results demonstrated that the combination therapy increased the percentage of objective responses (51). Another CCR2 inhibitor, CCX872, is really promising in the context of pancreatic tumors. In a preclinical setting, it improved the efficacy of the anti-PD-1 treatment (50) and positive results were also obtained in a clinical trial (NCT02345408) when used in combination with FX (53). In murine models of hepatocellular carcinoma (HCC), CCR2 targeting with the antagonists RDC018 or 747 in combination with Sorafenib, reduced tumor growth and metastasis with a corresponding decrease in macrophage infiltration (52, 54). In prostate and breast cancer, CCR2 was found expressed by tumor cells and to promote cancer growth and migration (98, 99). However, targeting CCL2 with the humanized monoclonal CCL2 neutralizing antibody CNTO 888 in a phase I trial (NCT00537368) in solid tumors and in a phase II trial (NCT00992186) in metastatic prostate cancer, was unsuccessful due to ineffectiveness of CNTO 888 in reducing CCL2 serum level (57, 58). More recent preclinical data indicated that in breast cancer models inhibition of CCL2 improved the response to radiotherapy (100) and was effective in preventing metastasis (56), but its discontinuation caused a rebound in the number of circulating monocytes increasing metastatic spreading. Finally, in ovarian cancer, a CCR2 inhibitor enhanced peptide vaccination (55). All these data suggest that targeting the CCL2-CCR2 axis could be effective especially in combination therapies but attention has to be given to fluctuations in the number of circulating monocytes that can produce controversial effects (56).

### CCR4

CCR4 is overexpressed in many hematologic malignancies such as Adult T-cell leukemia (ATL) and Cutaneous T-cell lymphoma (CTL). The human anti-CCR4 antibody Mogamulizumab eliminates tumor cells via antibody-dependent cellular cytotoxicity (ADCC) and is actually in use in Japan for the treatment of relapsed/refractory ATL (60). It is also considered the best therapy for previously treated CTL patients according to an international phase III trial (63). In addition, in preclinical studies, CAR-T cells generated against CCR4, were found effective in the treatment of a wide spectrum of T cell malignancies (59).

CCR4 is also considered a promising target for solid tumors for its activity in modulating leukocyte infiltrate, in particular for depleting T_regs_. In a preclinical model of renal cancer, Affi 5, a CCR4 blocking mAb, reduced tumor growth affecting the phenotype of myeloid cells and increasing the number of infiltrating NK cells (61). CCR4 is now considered a target for renal carcinoma patients (101). However, there are major concerns about the safety of the use of mAbs against CCR4 especially in patients previously subjected to allogenic bone marrow (BM) transplant. Anti-CCR4 mAbs are also depleting T_regs_ for few months, increasing the risk of graft-vs-host disease (102). For this reason, small molecule antagonists of CCR4 with less harmful side effects are in development and one of them, AF399/420/1802, considerably improved the efficacy of cancer vaccines in different preclinical tumor models (melanoma, lung, and colon cancer) by preventing T_regs_ induction (62).

### CCR5

The role of CCR5 in cancer remains still controversial; depending on the cell type on which it is expressed it can have a pro- or anti-tumoral role. When expressed by tumor cells it drives their growth and metastatization, while when expressed by T cells potentiates anti-tumoral responses (103). For instance in breast cancer, a dual role of the receptor has been reported in promoting antitumor immune responses, but being also associated with cancer progression and metastasis (104). More recent data indicate that CCR5 induces the mobilization of myeloid cells with pro-tumoral activity (105) and results obtained with preclinical and clinical models of colorectal cancer (CRC) indicate that targeting CCR5 with the negative allosteric inhibitor Maraviroc promoted the polarization of macrophages toward an antitumoral state. Very interestingly, objective partial response was reported in three out of five patients who received a combination of Maraviroc (NCT01736813) and chemotherapy (64). These data suggest that targeting CCR5 could have a major antitumoral effect on tumors that are CCR5 positive and have a prevalent myeloid infiltrate with immunosuppressive activity, while in other tumors CCR5 activity on T cells needs to be preserved for the correct development of the immune response.

### CCR7

The therapeutic application of CCR7 inhibitors is also extremely promising. CCR7 is overexpressed by many tumors driving both tumor growth and metastatization. By the use of siRNA technology, CCR7 inhibition resulted in decreased number of metastasis in a model of colon carcinoma (65) and inhibited the growth of prostate cancer (66). Moreover, reduction of CCR7 expression in breast cancer inhibited metastasis (106) and single-chain antibodies blocking CCR7 (MSM R707) were found able to inhibit brain metastasis of T-cell acute lymphoblastic leukemia (107).

### CXCR2

CXCR2 is expressed by many tumor cells and is involved in the chemotherapy resistance in different preclinical models of cancer. In breast cancer cells, CXCR2 deletion resulted in better response to Paclitaxel (68). In a melanoma model, the CXCR2 inhibitor Navarixin synergized with MEK inhibition (69) whereas, in an ovarian tumor model, the CXCR2 inhibitor SB225002 improved the antiangiogenic therapy Sorafenib (71). Finally, in human gastric cancer, Reparixin, a CXCR1 and CXCR2 inhibitor, enhanced the efficacy of 5-fluorouracil (72).

CXCR2 targeting inhibits tumor growth also because it affects myeloid cell infiltration. In pancreatic tumors, CXCR2 inhibition prevented the accumulation of neutrophils unleashing the T cell response (73), resulting in inhibition of metastatic spreading and improved response to anti-PD-1 (74). Interestingly, the combined treatment of CXCR2 and CCR2 inhibitors limited the compensatory response of TAMs, increased antitumor immunity and improved response to FX (75). Finally, in a prostate cancer model, CXCR2 inhibition by SB265610, decreased recruitment of myeloid cells and enhanced Docetaxel-induced senescence, limiting tumor growth (76).

Following these promising preclinical results, a phase II clinical trial with the CXCR2 inhibitor AZD5069 is ongoing in pancreatic cancer patients (NCT02583477). In addition, the safety of using Reparixin in combination with Paclitaxel was assessed (70) and a double-blind study with these drugs for metastatic triple-negative breast cancer is in progress (NCT02370238).

### CXCR4

The CXCR4 antagonist AMD3100 (Plerixafor) is clinically approved for the mobilization of hematopoietic stem cells (HSCs) for transplantation in patients with Non-Hodgkin's lymphoma (NHL) or MM (67). Beside the HSCs mobilization effect, many preclinical data and clinical trials with AMD3100 or other CXCR4 inhibitors are now suggesting their effectiveness in tumors.

Referring to hematological malignancies, some CXCR4 antagonists, like AMD3100 and the derivative AMD3465, enhanced the efficacy of conventional therapies inducing the mobilization of cancer cells from the protective environment of the BM. In murine models of AML, AMD3100 improved the efficacy of chemotherapy with Ara-C (77). Similar results were obtained in a phase I/II study in patients with relapsed AML (78). The CXCR4 antagonists LY2510924 was also able to suppress the proliferation and progression of AML used as monotherapy (79). Another CXCR4 antagonist, BKT140 had an anti-leukemic effect in a murine model of NHL and its action was synergic with Rituximab (82). Phase I trials are ongoing to evaluate the safety and tolerability of the anti-CXCR4 mAbs BMS-936564 in AML patients (NCT01120457) and PF-06747143 in hematological malignancies (NCT02954653) (83).

CXCR4 inhibitors have strong antitumor and anti-metastatic effects also in solid tumors. In glioblastoma (GBM), CXCR4 expression is higher in more aggressive tumors and is further upregulated by anti-angiogenic therapies (85). AMD3465 reduced the growth of xenografts of glioblastoma multiforme and medulloblastoma cell lines (108) and the CXCR4 antagonist PRX177561, increased the antitumor effects of Bevacizumab and Sunitinib in subcutaneous or orthotopic xenografts of glioblastoma models (91). The CXCR4 antagonist POL5551 inhibited GBM growth and dissemination after anti-VEGF therapy (86). Current clinical trials with AMD3100 in newly diagnosed or recurrent GBM patients are evaluating the safety and efficacy of daily subcutaneous injection (NCI2012-00149) or 2 weeks continuous intravenous infusion (NCI2013-02012). A phase I/II study of the CXCR4 antagonist USL311 alone and in combination with Lomustine is ongoing in patients with advanced solid tumors and relapsed/recurrent glioblastoma multiforme (NCT02765165).

In addition to brain tumors, AMD3465 and LY2510924 have been found to inhibit tumor growth and metastatization in many preclinical models (80, 84). LY2510924, tested in a phase I trial (NCT02737072), was found clinically safe and well-tolerated in advanced solid cancers (colorectum, lung, breast, and prostate) (81). A phase I trial (NCT01837095) of the CXCR4 antagonist Balixafortide plus Eribulin in HER2-negative metastatic breast cancer has given promising results (88).

Notably, CXCR4 inhibition is not only acting on tumor cells but is also promoting antitumoral T cell responses. In a pancreas tumor model, AMD3100, blocking the interaction of CXCR4 positive tumor cells with CXCL12 producing fibroblasts, unleashed a rapid accumulation of T cells and acted synergistically with anti-PD-L1 (89). In a mesothelioma model, AMD3100 increased the efficiency of the vaccine against mesothelin (VIC-008) by inhibiting PD-1 expression on CD8 T cells and by converting T_regs_ in T helper like cells (90). The inhibition of T_regs_ infiltration and the promotion of antitumoral T cell response by AMD3100 were also demonstrated in a mouse model of ovarian cancer (87).

## The Atypicals in the Immunotherapy Landscape

Atypical chemokine receptors (ACKRs) are emerging as crucial regulatory components of the chemokine network in a wide range of homeostatic and pathological conditions (109, 110). In this section, we reported preclinical observations and clinical data that provide evidences on their importance in cancer biology suggesting the possibility to validate them as new targets for innovative immunotherapies.

ACKR1 is mainly expressed on post-capillary and small collecting venular endothelial cells (ECs) and red blood cells (111), but also in many tumors such as GBM, hemangiosarcoma, erythroleukemia, breast, and colorectal cancers (112). It is able to bind a broad panel of both CC and CXC inflammatory chemokines acting as chemokine transporter. However, its role remains unclear in cancer because when expressed by ECs promotes tumor growth generating a chemokine gradient that sustains leukocyte infiltration (113). On the contrary, ACKR1 was reducing tumor growth in a model of prostate cancer (114) through the binding of angiogenic ELR^+^ CXC-chemokines that decreased angiogenesis and in a melanoma lung metastasis model, interacting with the tetraspanin CD82/KAI that induced tumor cells senescence (115). Finally, in breast carcinoma, ACKR1 expression correlated with a more favorable prognosis with less lymph nodes metastasis and better survival (116, 117).

ACKR2 plays a non-redundant role in the control of inflammatory response by scavenging and degrading most inflammatory CC chemokines, acting as agonists for receptors from CCR1 to CCR5 (118). It is expressed by trophoblast cells in placenta, lymphatic endothelial cells and at low levels by subsets of leukocytes (92, 119, 120). ACKR2 acts as a tumor extrinsic suppressor gene. Indeed, by dampening inflammation, it has a protective role in different inflammation-driven tumor models (121, 122). ACKR2 prevents tumor growth also when it is expressed by Kaposi's sarcoma cells where it is down-regulated by the oncogenic pathway KRAS/BRAF/MEK/MAPK (123), while in anaplastic thyroid carcinomas ACKR2 expression is downregulated by miR-146a (124). In both tumors ACKR2 downregulation unleashes pro-tumoral leukocyte infiltration.

On the contrary, ACKR2 has a tumor promoting role in the Apc-Min model of CRC limiting mast cells infiltration and activation of CD8^+^ T cells (125) and it has a pro-metastatic function in breast and melanoma cancer models, by limiting neutrophil and NK activity (92, 93).

ACKR3, is a high affinity receptor for CXCL12 and CXCL11 expressed by hematopoietic cells, mesenchymal cells, activated ECs, and neurons. ACKR3 negatively regulates CXCL11 and CXCL12 bioavailability and modulates CXCR4 expression and function (126, 127). In cancer, ACKR3 was found expressed on many tumor cells (such as renal carcinoma, breast cancer, and glioblastoma) and by tumoral ECs. It promotes tumor cell growth and metastasis (128, 129) acting on mTOR pathway (130). In lung adenocarcinoma, ACKR3 mediates TGF-ß1 promoted epithelial to mesenchymal transition (EMT) and tumor growth (131). ACKR3 is also expressed by aggressive prostate carcinoma cells (132) and in renal carcinoma patients with decreased survival and poor prognosis. In renal cell carcinoma, ACKR3 expressed by endothelial progenitor cells and tumoral ECs exerts a proangiogenic role inducing their migration and survival (133). In a glioblastoma murine model, mice treated with X7Ab against ACKR3 in combination with Temozolomide (TMZ) showed significant tumor reduction and longer survival, enhancing M1 macrophage activation (94).

The last member of the family, ACKR4 is a scavenger receptor for CCL19, CCL21, CCL25, and CXCL13. It is expressed by keratinocytes, thymic epithelium and bronchial cells (134). Some papers indicated a protective role of ACKR4 in tumors. In HCC tumors, it impaired chemotactic events associated with CCR7, limiting tumor progression and metastasis (135). ACKR4 down-regulation in human breast and colon cancer correlated with a worse outcome (136, 137). However, in breast carcinoma ACKR4 had a pro-metastatic role regulating EMT (138).

## Concluding Remarks

Being chemokines and chemokine receptors expressed by both tumor cells and leukocyte infiltrate they represent an ideal target for immunotherapy. However, better understanding of their roles in different malignancies is still necessary to avoid potential side effects. In hematological malignancies targeting of overexpressed chemokine receptors directly kill tumor cells but can potentially induce unwanted immune reactions (e.g., CCR4).

In the context of solid tumors, chemokine receptor inhibitors are giving encouraging results when used in combination with chemotherapy or with antibodies against immune checkpoints. For this reason, it is possible to envisage that chemokine receptor inhibitors will be used in the future to modulate the stromal component, to overcome chemotherapy resistance and to optimize the immune response of the patients.

## Author Contributions

VMP wrote the initial draft. AC, MM, and RB made substantial contributions and discussed the content. All authors reviewed and/or edited the manuscript prior submission.

### Conflict of Interest Statement

The authors declare that the research was conducted in the absence of any commercial or financial relationships that could be construed as a potential conflict of interest.
